# The Intersection of Metabolomics and Data Science

**DOI:** 10.3390/metabo13080915

**Published:** 2023-08-04

**Authors:** Seongho Kim

**Affiliations:** Biostatistics and Bioinformatics Core, Karmanos Cancer Institute, Department of Oncology, School of Medicine, Wayne State University, Detroit, MI 48201, USA; kimse@karmanos.org

Metabolomics generates a vast amount of data and heavily relies on data science for biological interpretation. By employing techniques from statistics, mathematics, computer science, and information science, data science aids in extracting valuable insights from large-scale metabolomics data. The Special Issue entitled ‘Data Science in Metabolomics’ focuses on data science applications in metabolomics and provides research articles and reviews that summarize major advancements and current challenges in this rapidly evolving field [1].

Traditional data analysis is predominantly centered on comparing the intensity values of features. However, intensity data can greatly vary due to factors such as different experimental batches, instruments, and pre-processing techniques or parameters [2]. Two novel approaches have been proposed to simplify intensity data using binary conversion [2,3]. Traquete et al. introduced binary simplification encoding for downstream analysis, including metabolic marker discovery [2]. Their method only considers the occurrence of spectral features by encoding feature presence and absence as binary. This approach performs consistently well, if not better, than traditional intensity-based methods. Kim et al. introduced the application of binary similarity measures in compound identification [3]. They illustrated the critical role of binary similarity measures in structure-based compound identification, demonstrating that the Fager–McGowan measure is more robust than the well-known Jaccard measure. Henglin et al. highlighted the importance of multivariate models for nontargeted metabolomics, particularly given the relatively small cohorts with a significant correlation between metabolites [4]. They demonstrated that sparse multivariate models exhibit robust statistical power and yield more consistent results.

Data science has made significant contributions to metabolomics by not only producing various open-source or commercial software packages but also by facilitating the sharing of experimental data and metadata through public data repositories. Many tools incorporate hundreds of functions and parameters for optimal data pre-processing, providing significant flexibility to experienced users but potentially overwhelming for inexperienced users. To enhance usability, even for occasional users, Nicolotti et al. streamlined the pre-processing of metabolomics mass spectrometry data and introduced an R workflow package, MStractor [5]. Powell and Moseley released an open-source Python package, ‘mwtab’, to improve curation and fairness for the Metabolomics Workbench (MW) repository [6]. The ‘mwtab’ package supports MW’s JSON-formatted analysis files, includes new validation functions for data deposition and meta-analyses, and offers extended functionality for interacting with non-‘mwTab’ MW data. These tools demonstrate the integration of data science techniques with metabolomics, enabling efficient data processing and advanced data interpretation.

The interaction between metabolomics and data science has led to numerous applications within the field of metabolomics. Davic and Cascio developed a microfluidic-laser-induced fluorescence system for detecting ultra-trace levels of primary fatty acid amines [7], and Kim et al. presented a comparative study of methods for controlling the false discovery rate in omics data analysis [8]. Sommariva et al. provided an in-depth review of the construction and numerical optimization of compartmental models in tracer kinetics for positron emission tomography [9]. Krishnan and Soldati-Favre focused on recent advancements in computational methods and high-throughput omics techniques used to study metabolic functions in the context of intracellular parasitism, with specific attention paid to human-infecting pathogens: Toxoplasma gondii and Plasmodium falciparum [10].

As the complexities of metabolomic data continue to increase, the role of advanced data science methodologies in unlocking its full potential becomes ever more pivotal.
